# Immune Checkpoint Inhibitors Combined with Oncolytic Virotherapy: Synergy, Heterogeneity, and Safety in Cancer Treatment

**DOI:** 10.32604/or.2025.067824

**Published:** 2025-11-27

**Authors:** Yi Feng, Haoxin Yang, Guicai Liang, Jun Chen, Tao Li, Yingjuan Wang, Jilin Chang, Yan Li, Meng Yang, Xilong Zhou, Zhiqiang Wang, Chunlei Ge

**Affiliations:** 1 Department of Cancer Biotherapy Center, Yunnan Cancer Hospital, The Third Affiliated Hospital of Kunming Medical University, Peking University Cancer Hospital Yunnan, Kunming, 650118, China; 2 Department of Laboratory Animal Science, Kunming Medical University, Kunming, 650500, China; 3 Department of Neurosurgery, Yunnan Cancer Hospital, The Third Affiliated Hospital of Kunming Medical University, Peking University Cancer Hospital Yunnan, Kunming, 650118, China; 4 Department of Radiology, Yunnan Cancer Hospital, The Third Affiliated Hospital of Kunming Medical University, Peking University Cancer Hospital Yunnan, Kunming, 650118, China; 5 Department of Radiation Oncology, First Affiliated Hospital of Kunming Medical University, Kunming, 650032, China

**Keywords:** Immune checkpoint inhibitors (ICIs), oncolytic virotherapy (OV), cancer immunotherapy, tumor microenvironment (TME)

## Abstract

Immune checkpoint inhibitor (ICI) has limited efficacy in the treatment of immune “cold” tumors. Due to insufficient T cell infiltration and heterogeneous programmed death ligand 1 (PD-L1) expression, the ORR is only 5%–8% compared with 30%–40% of “hot” tumors. This article reviews the synergistic mechanism, clinical efficacy and optimization strategy of oncolytic virus (OVs) combined with ICIs in the treatment of refractory malignant tumors. Systematic analysis of mechanistic interactions across tumor types and clinical trial data demonstrates that OVs transform the immunosuppressive microenvironment by inducing immunogenic cell death and activating innate immunity. Concurrently, ICIs enhance adaptive immunity by reversing T-cell exhaustion and expanding T-cell diversity. Clinical trials in melanoma, head and neck cancer and breast cancer showed superior efficacy. The Objective Response Rate (ORR) of combination therapy was 39%–62%, while the ORR of ICI monotherapy was 18%. Treatment heterogeneity is mainly attributed to virus-related factors, including targeting specificity and replication efficiency, tumor characteristics, such as antigen presenting ability and mutation load, and host immune status, including pre-existing antiviral antibodies and microbiome composition. This combined approach represents a paradigm shift in cancer immunotherapy, which effectively transforms immune “cold” tumors into “hot” tumors through the continuous activation of innate and adaptive immune responses. In the future, it is expected to improve the therapeutic effect of treatment-resistant malignant tumors through the integration of immune regulatory molecules, accurate biomarkers to guide the treatment scheme and triple combination strategy by a new generation of engineering viruses.

## 1 Introduction

### 1.1 Limitations of Immune Checkpoint Inhibitor (ICI) Monotherapy

**Figure 1:**
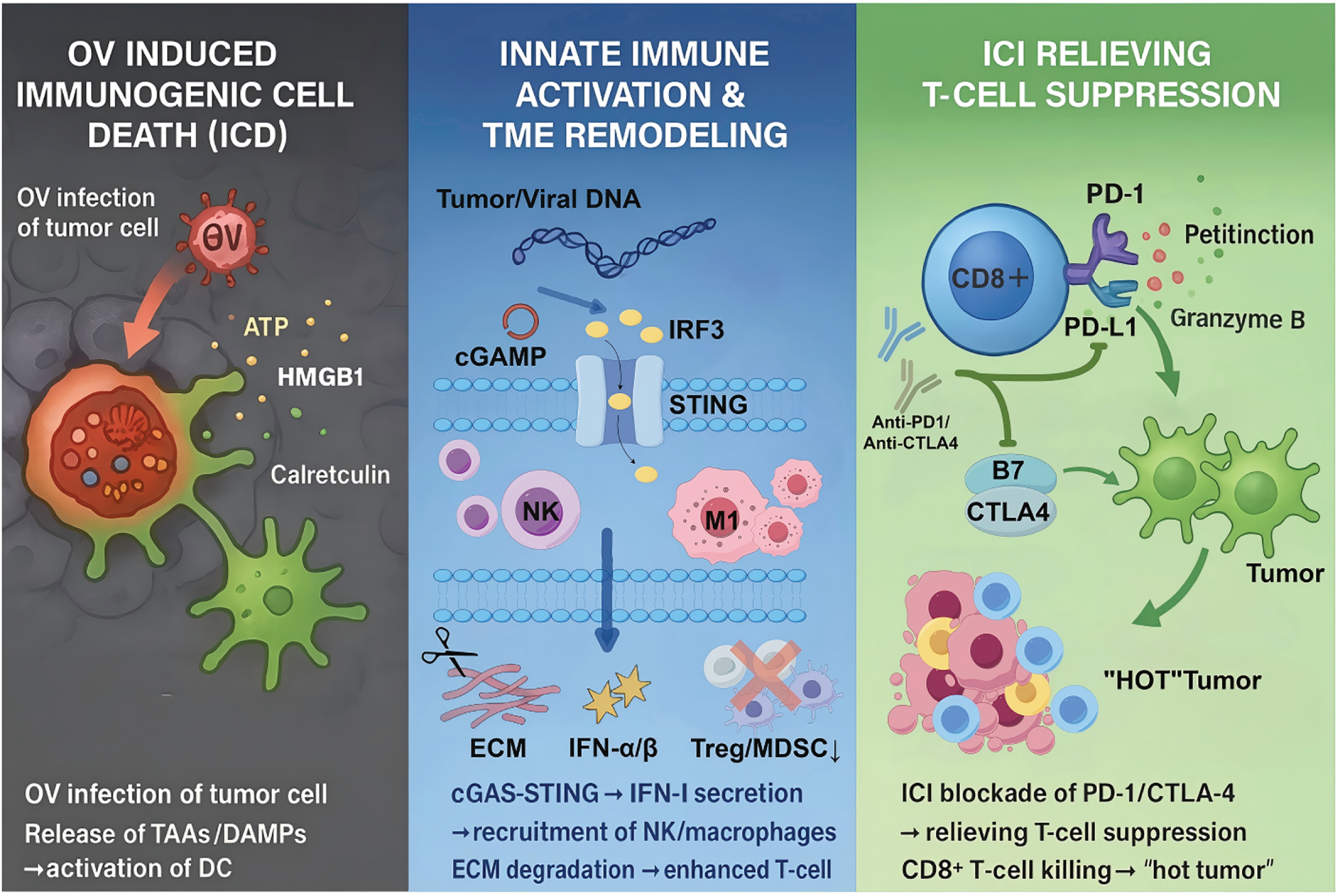
Synergistic anti-tumor mechanism of ICI and OV. OVs selectively infect and lyse tumor cells, releasing tumor-associated antigens (TAAs) and damage-associated molecular patterns (DAMPs), which activate dendritic cells (DCs) and recruit cytotoxic T lymphocytes (CTLs). Concurrently, ICIs block PD-1/PD-L1 or CTLA-4 pathways to reverse T-cell exhaustion, enhance clonal expansion, and sustain adaptive immune responses. This bidirectional interaction converts immunologically “cold” tumors into “hot” tumors, amplifying systemic antitumor immunity. Created with https://www.figdraw.com

Immune checkpoint inhibitors (ICIs) demonstrate significant limitations in solid tumor treatment, with therapeutic efficacy exhibiting substantial heterogeneity, which is directly related to the immunosuppressive characteristics of tumor microenvironment (TME) [1]. This heterogeneity is clinically classified by the “cold” and “hot” tumor framework, which is a conceptual model that classifies tumors according to pre-existing anti-tumor immunity and its internal response to ICIs. Immune “cold” tumors are characterized by less cytotoxic T lymphocyte (CTL) infiltration, antigen presentation defects, and immunosuppressive TME, which makes it difficult to tolerate ICI monotherapy. In contrast, the “hot” tumors usually show dense CD8^+^ T cell infiltration, active antigen presentation and pro-inflammatory TME, making them more vulnerable to checkpoint blocking (Table 1). Clinical evidence shows that immune “cold” tumors, including colorectal cancer with microsatellite stability, have an ORR of only 5%–8% against PD-1/PD-L1 therapy [1,2]. Pancreatic cancer showed a more limited response rate at 2%–3% [3,4]. In sharp contrast, the ORR of immune “hot” tumors (e.g., melanoma) was significantly higher than that of 30%–40% [5], while the response rate of highly microsatellite unstable tumors was 40%–50% [6,7]. This significant therapeutic heterogeneity is mainly due to insufficient infiltration of cytotoxic T lymphocytes (CTLs) in immune cold tumors, impaired antigen presentation mechanism characterized by reduced MHC-I expression, and heterogeneous expression pattern of PD-L1 [8,9]. Resistance mechanisms play a role at multiple biological levels through complex and dynamic interactions. At the tumor cell level, malignant cells evade immune recognition through epigenetic modifications, including abnormal DNA methylation patterns, activation of alternative immune checkpoint pathways (tim-3/lag-3) and inhibition of WNT/-catenin signaling cascade [10]. At the system host level, treatment resistance is characterized by T cell failure characterized by PD-1+TOX+phenotype, accumulation of regulatory T cells (Treg) and myeloid derived suppressor cells (MDSCs), and dysregulation mediated immunosuppression [11–13]. These multidrug resistance mechanisms together lead to limited clinical efficacy of ICI monotherapy, which requires the development of innovative combination therapy strategies (Fig. 1).

**Table 1:** The “Cold” vs. “Hot” tumor dichotomy in cancer immunotherapy

Feature	“Hot” Tumors	“Cold” Tumors	Reference
T-cell infiltration	High (CD8^+^ > 100 cells/mm²)	Absent/Low (CD8^+^ < 50 cells/mm²)	[1,8]
TMB	>10 mut/Mb	<5 mut/Mb	[1,2]
Key immune cells	CD8^+^ T cells, mature DCs	Tregs, MDSCs, M2 macrophages	[5,11]
PD-L1 expression	Homogeneous (≥1% CPS)	Heterogeneous/negative (<1% CPS)	[8,9]
Dominant signaling	IFN-γ, CXCL9/10	TGF-β, IL-10, VEGF	[14,15]
Stromal barriers	Low	High (fibrosis, hyaluronan)	[15]
ICI Monotherapy ORR	30%–50%	<10%	[1,2]
OV-ICI synergy target	Enhance T-cell function	Create T-cell influx	[16,17]

### 1.2 Unique Advantages of Oncolytic Virotherapy

OVs therapy has become a new strategy for tumor therapy because of its unique mechanism. OVs includes not only naturally occurring viruses with inherent tumor selectivity, but also genetically engineered variants designed to enhance tumor targeting and therapeutic efficacy. At present, the main virus platforms under clinical development include herpes simplex virus (HSV), adenovirus, vaccinia virus, reovirus and measles virus. Each virus has unique replication characteristics and immune stimulation characteristics. Although wild-type ov relies on natural tumor selective mechanisms, genetically modified armed variants carry immune regulatory genes that enhance and target immune responses in the TME [16]. Firstly, direct tumor lysis is its most important therapeutic property [16]. Genetically engineered viral vectors demonstrate tumor cell-specific infectivity and replication, achieving efficient cellular destruction while preserving normal tissue architecture. For instance, modified herpes simplex viruses and engineered vaccinia viruses (VVs) exclusively replicate within malignant cells, inducing cellular lysis through disruption of membrane integrity or metabolic pathway interference while maintaining the functional integrity of healthy tissues [18].

Second, ICD induced by OVs constitutes another major advantage. Viral infection of tumor cells triggers the release of damage-associated molecular patterns (DAMPs)—including Adenosine Triphosphate (ATP), high mobility group box 1 (HMGB1), and calreticulin—which activate dendritic cells and enhance tumor antigen presentation, eliciting specific CD8^+^ T-cell responses and transforming immunologically “cold” tumors into “hot” tumors [19,20]. For instance, OX40L-expressing oncolytic viruses significantly enhance anti-tumor immune memory by improving tumor antigen presentation [21].

Moreover, OVs successfully remodel the TME, resolving a significant drawback of traditional therapeutic strategies [22]. The TME usually consists of extracellular matrix (ECM) components, stromal components, and immunosuppressive cellular populations (MDSCs, Treg cells) that work together to prevent immune cell infiltration and therapeutic agent penetration [15,23]. Through cytokine secretion (IL-12, IFN-γ) and matrix component degradation, especially hyaluronan, OVs reverse this immunosuppressive environment. The specific pathway activation varies depending on the viral platform; RNA viruses such as reovirus activate different pattern recognition receptors, whereas HSV-based vectors primarily engage DNA sensing pathways [24,25]. Similarly, oncolytic vaccine viruses expressing hyaluronidase can break the hyaluronic acid barrier surrounding cancer tissues, making it easier for chemotherapeutic drugs and chimeric antigen receptor T (CAR-T) cells to penetrate cancer tissues. At the same time, they attract M1 polarized macrophages and natural killer cells to create a microenvironment with immune activity [26–28] (Fig. 1). Strategic pairing with ICIs or metabolic regulators (e.g., high-dose vitamin C) can enhance the therapeutic potential of OVs and produce synergistic antitumor effects [29].

### 1.3 The Scientific Hypothesis of Combination Therapy

The combination of OVs and ICIs shows significant synergistic potential in tumor immunotherapy, and its mechanism of action is complementary. OVs selectively infect and lyse cancer cells, releasing tumor antigens and danger signals that initiate innate immune system activation [16,20]. This cascade effect recruits and activates innate immune cells, including dendritic cells and macrophages, fundamentally transforming the immunosuppressive “cold” tumor phenotype into immunostimulatory “hot” tumors.

Specifically, oncolytic adenovirus system enhances the function of antigen presenting cells, promotes tumor specific T cell activation and clonal expansion, and creates the best conditions for ICI treatment intervention [30]. Subsequently, ICIs functions by neutralizing immunosuppressive signaling pathways, especially pd-1/pd-l1 and cytotoxic T lymphocyte antigen-4 (CTLA-4) axis, thereby reducing T cell inhibition and enhancing ov induced adaptive immune response (Fig. 1).

Empirical evidence shows that ov-ici combined therapy significantly increases the number, density and functional ability of tumor infiltrating CD8^+^ T cells, while weakening the immunosuppressive effect of Treg and myeloid derived suppressor cells (MDSCs), so as to effectively overcome the ICI resistance mechanism (which has been proved in preclinical models and clinical studies) [17,31]. This synergistic therapy represents a paradigm shift in cancer immunotherapy, which provides enhanced efficacy compared with single therapy through continuous and complementary activation of innate and adaptive immune responses.

## 2 Mechanistic Synergy

### 2.1 Reprogramming of the TME by OVs

#### 2.1.1 Virus-Induced Immunogenic Cell Death (ICD) and Antigen Release

OVs induce the death of immunogenic cells through selective infection and lysis of tumor cells, which is an important driving factor for the direct tumor clearance mechanism and TME remodeling. ICD promotes the exposure of calreticulin to the surface of tumor cells through endoplasmic reticulum stress characterized by reactive oxygen species accumulation, and promotes the release of HMGB1 and other DAMPs (including ATP) [32–34]. These molecules activate dendritic cells through pattern recognition receptors (e.g., toll like receptor 4 (TLR4) and interferon gene stimulating factor (STING)) to enhance the cross presentation of tumor associated antigens, thus recruiting and activating CD8^+^ T cells to establish tumor specific immune memory [17]. For instance, genetically modified oncolytic coxsackievirus B3 armed with bee venom peptide melittin and CpG oligodeoxynucleotide significantly augments DAMP release through melittin-mediated cell membrane disruption and CpG-dependent TLR9 activation in dendritic cells, enhancing immune cell infiltration and inhibiting both primary and distant tumor growth [35].

#### 2.1.2 Activation of Innate Immune Signaling

OVs remodel the immunosuppressive TME by activating innate immune signaling pathways, particularly the cyclic guanosine-adenylate synthase (cGAS)-STING pathway and type I interferon (IFN-I) secretion [36,37]. Following viral invasion of tumor cells, viral nucleic acids or cytoplasmic DNA generated by tumor damage are recognized by cGAS [38]. The enzyme catalyzes the production of the second messenger ring GMP-AMP (CGAMP), activates sting protein, triggers the signal axis of tank binding kinase 1 (TBK1)-interferon regulatory factor 3 (IRF3), and induces IFN-α/β secretion. For example, Newcastle disease virus activates IFN-I response through retinoic acid-induced gene I (RIG-I)/mitochondrial antiviral signal (MAVs) pathway, significantly enhancing antigen presentation and T cell infiltration [39,40]. Preclinical studies have shown that oncolytic adenovirus can up regulate the expression of IFN-I related genes in TME, and single cell sequencing confirmed that the expansion of CD8^+^ T cells is closely related to the activation of sting pathway [41]. It is worth noting that some tumors degrade cgamp by overexpression of exonucleotide pyrophosphatase/phosphodiesterase 1 (ENPP1) and inhibit sting signal, thus avoiding immune detection; OVs can reverse this immune escape mechanism and restore the antitumor activity of CGAs sting pathway. Genetically engineered viruses (e.g., adenoviruses carrying C-X-C motif chemokine ligand 10 (CXCL10) or poxviruses expressing TGF β inhibitors) can synergistically activate pattern recognition receptors, amplify IFN-I effects, and recruit cxcr3+immune cells to enhance anti-tumor response [14,42,43]. The combination of ICIs or radiotherapy can significantly enhance ifn-i-mediated immunogenicity and overcome the immunosuppressive disorder of TME [44].

#### 2.1.3 Promotion of T-Cell Infiltration

OVs effectively promote T-cell infiltration into the TME through genetic engineering to express chemokines (e.g., CXCL10, CCL5) or by disrupting physical tumor barriers, thereby enhancing anti-tumor immune responses [14]. Oncolytic adenovirus carrying CXCL10 significantly inhibits tumor growth and prolongs survival by continuously secreting CXCL10 and recruiting CD8^+^ T cells and NK cells expressing CXCR3 to the core tumor area [42]. In the mouse glioblastoma model, OVs expressing CXCL10 combined with PD-1 inhibitor not only promote T cell infiltration, but also reduce the proportion of Treg and M2 macrophages, reshaping TME into a pro-inflammatory state [43]. Similarly, OVs expressing CCL5 enhance the migration of T cells to tumors through CCR5 receptor binding, and reduce the physical barrier by degrading extracellular matrix components [45]. Oncolytic vaccinia virus ovv-hyal1 destroys the structure of tumor matrix by secreting hyaluronidase, releases chemotactic gradient signal, and promotes the localization of CAR-T cells and tumor infiltrating lymphocytes to the tumor core [26,45]. Preclinical studies have shown that chemokines combined with immunotherapy (for example, oncolytic adenovirus expressing chemokine ligand 11 (CXCL11) of the C-X-C motif combined with B7H3-CAR-T cells) can significantly enhance the therapeutic effect, indicating that the guidance of chemokines is the key strategy to overcome the immunosuppression of TME [46,47]. Future studies should optimize the combination of chemokines and drug delivery methods to maximize the activation of local immunity.

### 2.2 Enhancement of Adaptive Immunity by ICIs

#### 2.2.1 PD-1/PD-L1 Blockade Reverses T Cell Exhaustion

PD-1/PD-L1 signaling pathway represents the central regulatory mechanism of T cell depletion in TME. Under chronic antigen stimulation, tumor specific CD8^+^ T cells continue to overexpress PD-1, leading to functional inhibition and epigenetic reprogramming, which is characterized by loss of effector function, reduced proliferation and co expression of multiple inhibitory receptors, such as T cell immunoglobulin and mucin domain containing protein 3 (Tim-3) and lymphocyte activating gene 3 (LAG-3) [48,49]. PD-1/PD-L1 blockade reverses T-cell exhaustion by relieving TCR signaling inhibition, restoring cytotoxicity and cytokine secretion capacity [48]. Preclinical studies have shown that anti-PD-1 antibody promotes the transformation of failed T cells into functional effector T cells, and down regulates failure related genes (e.g., thymocyte selection related HMG box protein (tox), nuclear receptor subfamily 4a (NR4A)) [50,51]. In addition, the combination of interleukin-2 (IL-2) therapy induces stem cell like depleted T cells to differentiate into high affinity IL-2 receptor (CD25^+^) effector T cells by activating STAT5 signal, thereby reshaping the anti-tumor immune response and further enhancing the blocking effect of PD-1 [52]. It is worth noting that single cell TCR sequencing confirmed that CTLA-4 inhibitor combined with PD-1 blocker synergistically enhanced the temporal and spatial dynamics of T cell clones, significantly improving the breadth and persistence of anti-tumor immunity [53].

#### 2.2.2 CTLA-4 Inhibitors Expand T Cell Repertoire Diversity

CTLA-4 inhibitors expand the diversity of the initial T cell pool and enhance the anti-tumor immune response by targeting the co inhibitory signal at the early T cell activation stage and destroying the competitive inhibition of the CD28 co-stimulatory pathway [54]. Studies have shown that CTLA-4 blocking significantly increases the clonal diversity of T cell receptors in peripheral blood and TME, especially promoting the activation and expansion of low abundance tumor antigen-specific T cells [55]. In patients with metastatic melanoma, the increase of TCR β chain diversity caused by anti-CTLA-4 treatment was positively correlated with clinical efficacy. In terms of mechanism, CTLA-4 inhibitor indirectly enhances the expansion of effector T cell clone by inhibiting the immunosuppressive function of regulatory T cells [56]. Preclinical models show that CTLA-4 blockade can trigger a dynamic feedback loop between regulatory T cells and dendritic cells: regulatory T cells limit the co stimulatory ability of dendritic cells by down regulating B7 molecules dependent on CTLA-4, while CTLA-4 blockade can disrupt this balance and promote dendritic cell-mediated T cell activation and clonal diversity [57,58]. Notably, single-cell TCR sequencing confirms that CTLA-4 inhibitors combined with PD-1 blockade synergistically enhance T-cell clone spatiotemporal dynamics, significantly improving anti-tumor immunity breadth and durability [57].

### 2.3 Key Molecules in Bidirectional Regulation

#### 2.3.1 Mechanisms of OV-Induced PD-L1 Upregulation and the Synergistic Necessity of ICI

Additionally, epigenetic mechanisms may contribute significantly to this process, though the extent and context-dependency of these relationships require further investigation. Emerging evidence suggests that OV-induced PD-L1 upregulation may be coupled to epigenetic remodeling, though this association appears to be tumor type-specific and context-dependent. OVs potentially trigger DNA damage responses (e.g., Ataxia Telangiectasia Mutated/Ataxia Telangiectasia and Rad3-related protein (ATM/ATR) pathway activation) through viral replication-associated genomic stress [59], leading to histone H3 lysine 4 trimethylation (H3K4me3) enrichment at the PD-L1 promoter region [60]. Potentially promoting proteasomal degradation of DNA methyltransferase 3B [61]. This process could result in PD-L1 promoter hypomethylation, thereby potentially derepressing transcriptional activity.

Although OV-induced PD-L1 upregulation serves as an indicator of successful viral infection and often correlates with enhanced viral replication within tumor tissues, the resulting T-cell exhaustion and immune checkpoint activation severely limit anti-tumor immunity persistence. Combined application of ICIs achieves bidirectional regulation by targeting the PD-1/PD-L1 axis: PD-L1 blockers (e.g., atezolizumab) remove T-cell inhibitory signaling, restoring CD8^+^ T-cell proliferative capacity and cytotoxic function while prolonging immunological memory effects [53]. Simultaneously, ICIs reduce immune clearance of OVs, promoting viral spread and continuous replication in tumor tissues. Preclinical studies reveal that adaptive PD-L1 upregulation in local and distant tumors following oncolytic virus monotherapy can be effectively reversed by ICI blockade, significantly inhibiting untreated distant tumor growth and highlighting combination therapy’s “abscopal effect” advantage [62–64]. This synergistic strategy initiates immune responses via OVs while maintaining T-cell activity through ICIs, creating a positive cycle of anti-tumor effects. Future studies should explore drug delivery optimization and epigenetic interventions to overcome existing therapeutic limitations.

#### 2.3.2 Viral Vector Engineering Strategies

Engineering of OVs represents a key strategy to enhance their immunomodulatory function. Through gene editing techniques, OVs can serve as delivery vectors carrying immunomodulatory molecules (e.g., cytokines or ICIs) [65]. This allows local release of high concentrations of bioactive molecules in the TME, remodeling immunosuppressive conditions and enhancing anti-tumor immune responses. For example, Talimogene Laherparepvec (Oncolytic Herpes Simplex Virus-1 expressing GM-CSF) (T-VEC) [18], the first approved oncolytic virus therapy, through inducing tumor cell lysis, then releasing GM-CSF, promoting dendritic cell maturation and antigen presentation, thus activating tumor specific T cells, has shown significant efficacy in the treatment of melanoma (Optim Test showed that the target effective rate was 31.5%) [66,67].

Engineered OVs can directly express ICIs to block immunosuppressive signals. For instance, vaccinia virus cf-33-hnis-antipd-l1 expressing anti-PD-L1 monoclonal antibody can significantly reduce the tumor burden and prolong the survival time of colorectal cancer model by locally blocking PD-1/PD-L1 axis, activating CD8^+^ T cells and reducing myeloid derived suppressor cell infiltration [68,69]. Similar strategies include HSV-1 oncr-177, which induces lasting anti-tumor immune memory in various immune active tumor models by encoding anti-PD-1 and anti-CTLA-4 single chain variable region fragments [70,71].

Local cytokine delivery (e.g., IL-12) represents another important direction. Interleukin-12 (IL-12) promotes Th1 type immune response and enhances the activity of natural killer cells and cytotoxic T lymphocytes [16]. VG161is a HSV-1 carrying IL-12, IL-15 and PD-L1 blocking peptides. By reversing immunosuppressive TME, it significantly increases the number of tumor infiltrating lymphocytes and the sensitivity of PD-1 inhibitors in liver cancer models [72,73]. Similarly, vaccinia virus expressing an Interleukin-23 (IL-23) variant induces systemic anti-tumor immunity in melanoma models by activating T cells and promoting IFN-γ secretion [74,75].

The advantage of these engineering strategies is to continuously release local high concentrations of immune regulatory molecules through virus replication, avoid systemic toxicity, and synergistically enhance the direct cytotoxic effect and immune activation function of OVs. In the future, methods involving multi gene co expression or combined with existing immunotherapy are expected to overcome the limitations of solid tumor treatment.

## 3 Progress in Clinical Research (Clinical Evidence)

### 3.1 Melanoma

The NCT01740297 clinical trial represents the first randomized controlled study investigating the safety and efficacy of talimogene laherparepvec (T-VEC) plus ipilimumab for advanced unresectable melanoma. In the phase Ib study, the ORR among 18 evaluable patients reached 56%, including a complete response rate of 33%, substantially exceeding historical data from either agent alone [76]. The subsequent phase II study (n = 198) further confirmed that the T-VEC plus ipilimumab group demonstrated significantly higher ORRs compared to ipilimumab monotherapy (39% vs. 18%, *p* = 0.002) with more durable responses [66]. Long-term follow-up data revealed a median duration of response of 69.2 months in the combination arm, with 18-month progression-free survival and overall survival rates of 50% and 67%, respectively [77,78]. In addition, in a study of masterkey-265, a clinical trial of T-VEC combined with pembrolizumab in the treatment of advanced melanoma. In the phase 1b part of the study, a total of 21 patients with advanced melanoma received T-VEC plus pembrolizumab, achieving an ORR of 61.9% and a CR of 33.3% [77,78]. Regarding safety, the adverse event profile of the combination was consistent with the individual agents, with grade 3/4 treatment-related adverse events occurring in 26.3% of patients, with no unexpected safety signals observed. Notably, the combination therapy demonstrated antitumor activity not only in injected lesions but also in distant uninjected sites including visceral metastases, indicating that this strategy can induce systemic antitumor responses through local immune activation. These findings provide compelling clinical evidence for the combination of oncolytic virotherapy with immune checkpoint inhibition, offering a novel treatment option for patients with advanced melanoma. Other promising OV-ICI combinations include a phase II study (NCT02272855) demonstrating that HF10 (naturally attenuated HSV-1) combined with ipilimumab treated 46 advanced melanoma patients with an immune-related ORR of 41% (immune-related CR 18%) and regression in 52% of uninjected lesions [79]. Challenges remain in targeting rare subtypes (mucosal and acral melanomas) with their characteristically low tumor mutational burden and limited response to ICI monotherapy (ORR of only 15.2% for PD-1 inhibitors alone) [80,81]. In the phase III OPTiM trial, T-VEC significantly increased the durable response rate compared with GM-CSF (16.3% vs. 2.1%) and improved median overall survival (23.3 months vs. 18.9 months) [82]. Emerging strategies include neoadjuvant OV-ICI therapy protocols and novel engineered viruses. Although the phase III T-VEC-pembrolizumab study did not meet its primary survival endpoint, the improved response rates emphasize the promise of OV-ICI combination therapy in biomarker-defined subgroups (Table 2).

**Table 2:** Clinical trials of OV combined with ICIs

Oncolytic viruses	Immune checkpoint inhibitors	Indication	Response date	Clinical trials gov identifier
T-VEC	Ipilimumab	Melanoma	ORR 39% (T-VEC + ipi) vs. 18% (ipi); *p* = 0.002	NCT01740297
T-VEC	Pembrolizumab	Stage IIIB–IV melanoma	48% ORR	NCT02263508
T-VEC	Pembrolizumab	HNSCC	ORR 16.7% and disease control rate 38.9%	NCT02626000
T-VEC	Pembrolizumab	HCC, liver metastases	N/A	NCT02509507
CACATAK	Pembrolizumab	Melanoma	N/A	NCT02565992
CACATAK	Pembrolizumab	NSCLC and bladder cancer	N/A	NCT02043665
HF10	Ipilimumab	Melanoma	N/A	NCT03153085
HF10	Ipilimumab	Melanoma	BORR at 24 weeks 41%; median PFS 19 months; median OS 21.8 months	NCT02272855
VSV-IFNβ-NIS	Pembrolizumab	NSCLC and HCC	N/A	NCT03647163
H101	Camrelizumab	Bladder Cancer	N/A	NCT05564897
ONCOS-102	Pembrolizumab	Melanoma	ORR 35%	NCT03003676
TILT-123	Pembrolizumab	Platinum-refractory Ovarian Carcinoma	N/A	NCT05271318
DNX-2401	Pembrolizumab	Glioblastoma	ORR10.4% 12 months OS 52.7%	NCT02798406
VB-111	Nivolumab	Colorectal Cancer	N/A	NCT04166383

Note: HNSCC, head and neck squamous cell carcinoma; HCC, hepatocellular carcinoma; NSCLC, non-small cell lung cancer; TNBC, triple negative breast cancer; ORR, objective response rate; BORR, best objective response rate; OS, overall survival; N/A, not available; PFS, progression-free survival. Data were collected from National Clinical Trials (https://clinicaltrials.gov/).

### 3.2 Head and Neck Squamous Carcinoma (HNSCC)

In HNSCC treatment, several clinical trials have explored OV-ICI combination strategies with promising results. A phase Ib study evaluated T-VEC in combination with pembrolizumab in 36 patients with platinum-refractory recurrent/metastatic HNSCC. This combination demonstrated a manageable safety profile (10% incidence of grade 3–4 adverse events), with an ORR of 16.7%, median progression-free survival of 3.0 months, and overall survival of 5.8 months [83]. Tumor regression correlated with increased immune infiltration, suggesting potential for enhanced efficacy through optimization of viral vectors [84].

Current phase I trials are investigating engineered HSV-1 virus ONCR-177 (carrying multiple immunomodulatory genes) in combination with pembrolizumab [83,85]. A phase II/III study of Reolysin (reovirus) with paclitaxel/carboplatin showed that while phase III trials did not meet the primary endpoint, HPV-negative subgroups demonstrated clinical benefit, highlighting the importance of biomarker-guided patient selection [86,87].

Key challenges include insufficient response rates in tumors with low PD-L1 expression, limited viral delivery efficiency, and heterogeneous patient responses. Future directions should focus on engineered viruses, combination with radiotherapy, and optimizing patient stratification using biomarkers such as HPV status and T-cell clonality (Table 2).

### 3.3 Breast Cancer

Vaccinia virus-based studies have demonstrated promising clinical activity in breast cancer. The phase I/IIa trial (NCT04725331) evaluated intratumoral Pexa-Vec (VV) combined with pembrolizumab in advanced solid tumors, including breast cancer. Preliminary data showed enhanced immune infiltration and reduced tumor burden in triple-negative breast cancer (TNBC) models, and under the combination therapy, 2 of the 6 patients who failed previous monotherapy experienced clinical response [88,89]. Another phase II trial (NCT02630368) using VV with cyclophosphamide and avelumab in advanced breast cancer demonstrated prolonged progression-free survival and significantly upregulated immune-related gene expression in human epidermal growth factor receptor 2-positive (HER2^+^) and TNBC subtypes [89,90]. Reovirus-based therapies have further expanded combination strategies. A phase II trial evaluating intravenous pelareorep (reovirus) with anti-PD-1 antibody in TNBC demonstrated reduced metastatic burden and increased anti-tumor immunity [89]. Another phase II trial combining pelareorep, avelumab, and paclitaxel in hormone receptor-positive/HER2-negative metastatic breast cancer significantly prolonged survival compared to monotherapy [18]. A notable case study reported that a patient with HER2-positive recurrent breast cancer achieved tumor volume reduction from 2.47 to 0.91 cm³ through sequential intratumoral injections of measles virus and vesicular stomatitis virus, with no recurrence at 45-month follow-up [88].

Despite promising early results, clinical translation faces challenges including delivery efficiency limitations, tumor heterogeneity effects, and potential immunotherapy-related toxicities. Nanocarrier encapsulation and magnetically guided delivery technologies are being explored to enhance targeting precision [91]. Biomarker development and dynamic monitoring remain essential for accurate patient stratification and therapy optimization (Table 2).

### 3.4 Analysis of Efficacy Heterogeneity

#### 3.4.1 Commonality of Positive Results

The efficacy of ICI combined with OV therapies demonstrates significant heterogeneity among different cancer types and patient populations. Analysis of successful combination studies reveals several recurring factors that contribute to positive therapeutic outcomes, though the relationships between these factors and efficacy are more complex than initially apparent.

① Tumor Mutational Burden and Neoantigen Release

Tumors with high tumor mutational burden (TMB) have demonstrated enhanced responses to OV-ICI combinations, though the evidence base specific to oncolytic virotherapy remains limited compared to broader immunotherapy studies. In melanoma combination studies, patients with TMB > 10 mutations/megabase showed superior ORRs compared to those with lower mutational loads [77,78]. However, the mechanistic contribution of high TMB to OV efficacy specifically requires further investigation, as OVs may overcome the limitations of low neoantigen burden through alternative pathways including DAMP release and viral antigen presentation [17,31].

The release of tumor-associated antigens through virus-induced oncolysis provides a mechanism that may be partially independent of baseline mutational burden. When T-VEC was combined with pembrolizumab in melanoma patients, ORRs reached 61.9%, representing improvement over monotherapy regardless of initial TMB status, suggesting that viral-mediated antigen release can complement endogenous neoantigen presentation [78].

② Viral Replication Efficiency: Balancing Oncolysis and Transgene Expression

The relationship between viral replication efficiency and therapeutic outcomes presents a more complex dynamic than previously characterized. While efficient viral replication enhances direct oncolytic effects and DAMP release, the temporal kinetics of transgene expression must be carefully considered in the context of combination therapy objectives. Most transgene-expressing OVs, including T-VEC (GM-CSF), require active viral replication for transgene production. Paradoxically, excessively rapid viral replication and subsequent cell lysis may truncate the duration of immunomodulatory factor expression, potentially limiting the establishment of sustained antitumor immunity [92,93]. Clinical evidence suggests that an optimal temporal window exists where viral replication provides sufficient transgene expression duration while maintaining effective oncolytic activity. For example, engineered herpes simplex viruses with attenuated replication kinetics have demonstrated prolonged transgene expression compared to rapidly replicating variants, resulting in enhanced CD8^+^ T cell recruitment and improved combination efficacy with checkpoint inhibitors [94]. This suggests that therapeutic optimization may require balancing replication efficiency with transgene expression sustainability rather than maximizing viral replication alone.

③ Baseline Immune Infiltration and Microenvironment Characteristics

Pre-existing CD8^+^ T cell infiltration remains a consistent predictor of combination therapy success. Patients with baseline CD8^+^ T cell density ≥100 cells/mm² demonstrated significantly higher objective response rates with OV-ICI combinations compared to those with lower infiltration levels [94]. The presence of functional antigen-presenting machinery, including adequate MHC-I expression and intact antigen processing pathways, correlates with enhanced viral antigen cross-presentation and subsequent T cell activation [93,95].

However, there are cases where OVs did not significantly improve ICI efficacy. In the JAVELIN Merkel 200 trial, avelumab (anti-PD-L1) with T-VEC for advanced Merkel cell carcinoma failed to significantly improve progression-free survival, with only a 9% ORR improvement over monotherapy [96]. Potential reasons for treatment failure include:

① TME Suppression: High proportions of Regulatory T cells (Tregs) and M2-type macrophages can counteract OV-induced immune activation through TGF-β and IL-10 secretion.

② Inadequate Viral Targeting: T-VEC depends on herpesvirus entry receptor (HVEM) expression; low HVEM expression leads to inefficient viral replication.

③ Immunosuppressive Stroma: Tumor-associated fibroblasts form physical barriers to T-cell infiltration by secreting fibronectin.

Common factors in other negative outcomes include:

① Antigen Presentation Defects: Low MHC-I expression or β2-microglobulin mutations result in failure to present antigens to T cells.

② Host Preexisting Immunity: Antiviral neutralizing antibodies can rapidly clear OVs and diminish efficacy.

③ Non-Redundant Resistance Mechanisms: Tumors may escape immune recognition by upregulating alternative immune checkpoints (e.g., LAG-3, TIM-3) or activating the WNT/β-catenin pathway.

## 4 Sources of Heterogeneity in Efficacy

### 4.1 Virus-Related Factors

Oncolytic virus targeting specificity constitutes a critical determinant of efficacy heterogeneity when combined with ICIs, encompassing complex multilevel virus-host interaction regulatory networks. At the initial target recognition level, viral tropism is defined by the molecular complementarity between viral receptor-binding proteins and specific tumor surface markers: Enterovirus A71(EV-A71) selectively infects glioma cells via the Scavenger Receptor Class B Member 2 (SCARB2) scavenger receptor, whereas wild-type HSV relies on NECTIN-1/HVEM receptor-mediated cell entry [97,98]. Menotti’s team inserted interleukin-13 (IL-13) or single chain Fv (scFv) into HSV glycoprotein gD to promote the recognition of tumor antigens (e.g., her2/egfr), thereby increasing the specificity of virus infection by 4–6 times, which indicates that this specificity can be enhanced through genetic engineering strategies [99]. To enhance targeting precision, researchers have developed dual regulatory systems that combine tissue-specific microRNA response elements (e.g., brain tissue miR124) with receptor engineering, resulting in 8-fold enhancement of viral replication efficiency within target organs [100].

At the host defense level, OVs must overcome both intrinsic cellular immune barriers and immunosuppressive TMEs to achieve optimal efficacy. Hong et al. demonstrated that protein kinase R (PKR) not only inhibits viral replication through Eukaryotic Initiation Factor 2α (eIF2α) phosphorylation but also establishes immunosuppressive barriers via TGF-β signaling pathway activation [36]. Their engineered oHSV-shPKR virus increased viral titer 3.8-fold by silencing PKR expression and enhanced CD8^+^ T-cell infiltration from 12% to 34%, confirming the synergistic therapeutic effect of targeting host defense mechanisms [36]. The innovation of delivery strategy significantly affects the target efficiency; It is worth noting that Vannini’s team used mesenchymal stromal cells to encapsulate HSV virus particles. By regulating the heparin sulfate interaction, the cycle half-life was prolonged by 2.4 times and the tumor virus load was increased by 4.7 times [101]. This complex drug delivery system migrates to the metastasis through chemokine receptors, promotes space-specific infection, and expands the treatment scope of oncolytic virus therapy.

The difference of virus targeting mechanism directly affects the subsequent immune activation mode: specifically, scarb2 targeting ev-a71 mainly induces type I interferon response, while EGFR engineered HSV drives helper T cell 1 (Th1) cell polarization through cytokines such as CCL5 and IL-12 [99,102]. When combined with PD-1 inhibitors, the choice of targeting strategy significantly affects combination efficacy; in a hepatocellular carcinoma model, granulocyte-macrophage colony-stimulating factor (GM-CSF)-carrying targeted viruses combined with anti-PD-L1 therapy increased objective response rates from 24% to 61%, though exclusively in PD-L1-positive subgroups [36,99]. These findings underscore the necessity to model associations between viral targeting characteristics and immune checkpoint expression profiles to achieve precise prediction and optimization of combination therapy efficacy through multi-omics-guided personalized viral design and comprehensive biomarker stratification.

**Figure 2:**
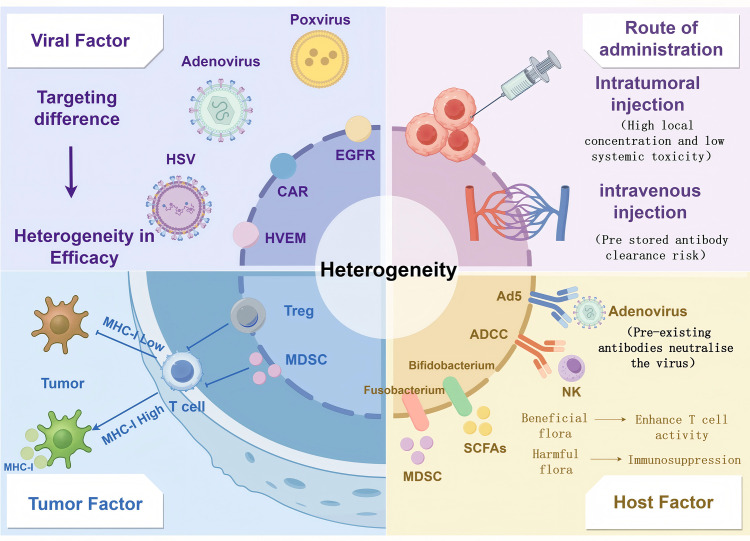
Multifactorial modelling of heterogeneity in OV-ICI efficacy. Virus-related factors: Including viral tropism (e.g., receptor specificity), replication efficiency, and delivery strategies (intratumoral vs. systemic administration). Tumor-related factors: Encompassing antigen presentation defects (e.g., MHC-I downregulation), immunosuppressive stromal components (Tregs, MDSCs, and fibrotic barriers), and tumor mutational burden (TMB). Host-related factors: Such as preexisting antiviral neutralizing antibodies, gut microbiota composition, and systemic immune status. Created with https://www.figdraw.com

The drug delivery route significantly affects viral distribution in tumor tissues and efficacy heterogeneity. Intratumoral injection, the most commonly used delivery mode, directly delivers the virus to the tumor sites, reducing systemic exposure risks and enhancing tumor lysis through high local viral replication concentration [103]. However, this method has great limitations: it is difficult to accurately locate deep or hard to reach tumors (e.g., pancreatic cancer and glioma), and repeated injection may increase the risk of bleeding or metastasis [104]. Additionally, intratumoral injections depend on the technician’s experience, limiting treatment accessibility and consistency. In contrast, intravenous injection allows systemic virus distribution and may target metastatic tumors, but the virus is easy to be cleared by circulating neutralizing antibody, and is limited by abnormal tumor vascular structure, resulting in insufficient virus enrichment in TME [105]. For example, after intravenous injection, adenovirus tends to accumulate in the liver, which limits the targeting efficiency [106]. Recent strategies to enhance targeting and evade immune clearance include engineered modified viral capsids (e.g., FX-binding-deficient Ad5) or cellular vectors (e.g., mesenchymal stem cell-loaded OVs), which have demonstrated promotion of sustained viral replication at tumor sites and activation of systemic antitumor immunity [107,108]. However, intravenous administration still faces challenges such as short virus half-life and targeted toxicity. Future progress requires new delivery technologies (e.g., nano carriers, genetic engineering) to optimize systemic virus delivery and tumor specific enrichment [109] (Fig. 2).

### 4.2 Tumor-Related Factors

Defective antigen presentation and immunosuppressive cell dynamics are the key biological barriers for effective combination therapy. At the level of antigen presentation, about 40%–60% of solid tumors showed down-regulation or dysfunction of MHC-I molecule, mainly due to β 2-microglobulin (B2M) gene mutation and epigenetic silencing of antigen processing related transporter (TAP) [110]. This defect impedes efficient presentation of TAAs released by OVs to CD8^+^ T cells, resulting in what can be characterized as “antigenic visibility loss”. Notably, preclinical models demonstrate that OVs can induce tumor cells to re-express MHC-I and enhance dendritic cell cross-presentation by carrying immunomodulatory genes such as GM-CSF or interferon-gamma (IFN-γ), exemplified by T-VEC’s GM-CSF engineering. When administered concomitantly, anti-CTLA-4 antibodies synergistically promote lymph node T cell priming, thereby partially overcoming local antigen presentation defects [82,84,94] (Fig. 2).

Reprogramming immunosuppressive TME is another major challenge for combination therapy. New data show that in the early stage of combined therapy, Tregs are transiently amplified, which may inhibit the Th1 immune response induced by oncolytic virus through dual CTLA-4 and lymphocyte activating gene 3 (LAG-3) inhibitory signaling pathway [16,99]. MDSC dynamics exhibit greater heterogeneity; adenoviral vectors induce C-X-C motif chemokine ligand 1 and 2 (CXCL1/CXCL2)-mediated polymorphonuclear-MDSC (PMN-MDSC) infiltration, whereas herpesvirus vectors promote monocytic-MDSC (M-MDSC) differentiation through toll-like receptor 9 (TLR9) activation [111,112]. Clinical studies have demonstrated significantly higher objective response rates (ORR 3.2, 95% CI 1.8–5.7) in patients with ≥50% decrease in peripheral blood regulatory T cell to effector T cell (Treg/Teff) ratios at week 3 of treatment when anti-PD-1 antibodies combined with talimogene laherparepvec (T-VEC) were administered for melanoma, highlighting the importance of systemic immune monitoring [113]. Recent strategies involving the expression of C-C motif chemokine ligand 5 (CCL5) and C-X-C motif chemokine ligand 10 (CXCL10) in engineered OVs can selectively recruit effector T cells and physically segregate regulatory T cell-myeloid-derived suppressor cell (Treg-MDSC) clusters, offering innovative approaches for tumor microenvironmental remodeling [114–116].

### 4.3 Host-Related Factors

#### 4.3.1 Impairment of Efficacy by Preexisting Antiviral Antibodies

The pre-existing antiviral antibodies (neutralizing antibodies, NABs) in the host are the key determinants of the heterogeneity of oncolytic viral therapy. The emerging evidence reveals a complex two-way relationship, which goes beyond the previously assumed one-way inhibitory effect, and proposes a mechanical paradox that requires a comprehensive examination of this complex immune interface [117].

The predominant mechanistic understanding suggests an inhibitory role for neutralizing antibodies: for adenoviral vectors, adenovirus serotype 5 (Ad5)-specific antibodies, present in approximately 60% of the population, block viral binding to the coxsackie-adenovirus receptor (CAR) and accelerate viral clearance by targeting the hypervariable region (HVR) of the viral capsid and the fibronectin structural domain [118,119]. Preclinical studies have quantitatively demonstrated that Ad5 antibodies reduce the viral load in the tumor by 70%–90%. This suppression effect is reflected clinically, as evidenced by the significantly shorter overall survival of patients with high antibody titers compared to those with low titers in the Delta-24-RGD glioma study (median overall survival 14.3 vs. 25.1 months, *p* = 0.032) [120].

Most strikingly, survival almost doubled in herpes simplex virus type 1 (HSV-1) seropositive patients treated with CAN-3110 compared to seronegative patients (median overall survival 11.6 vs. 6.0 months, hazard ratio = 0.49, *p* = 0.024) [120]. This apparent survival benefit contradicts the neutralization paradigm and can be explained by three potential mechanisms: (1) antibody-dependent cellular cytotoxicity (ADCC) through the binding of antibody-coated infected cells to natural killer (NK) cells; (2) dendritic cell receptor-mediated viral antibody complex uptake enhances antigen presentation efficiency; (3) modulation of inflammation through activation of the complement cascade, as demonstrated by elevated complement component 3b (C3b) and complement component 5b (C5b) levels in seropositive patients [121].

Whether inhibition or enhancement dominates may depend on specific factors of the situation; The research on Newcastle disease virus by MEDI5395 provides important insights into this background dependence. In patients with high CD8^+^ T cell density and PD-L1 positivity, although all patients had high antibody titers, the objective remission rate was 10.3% [117]. This indicates that favorable immune microenvironment features can overcome the neutralizing effect of NAb. This result is consistent with the mouse model, indicating that the pre-existing immune suppression of smallpox virus suppresses virus replication, but when combined with checkpoint inhibitors, immune activation is enhanced, paradoxically enhancing anti-tumor effects [118].

The virus structure and the method of administration also influence the effect of NAb. For non-enveloped viruses (e.g., adenoviruses), complement-anchored antibodies (IgG1, IgG3), which facilitate clearance, predominate, whereas for enveloped viruses such as HSV-1, non-complement-anchored antibodies (IgG4) are more common, which explains the different results Potential [122,123]. Intravenous administration is more prone to NAb neutralisation than intra-tumor administration, with studies demonstrating an 85% reduction in systemic viral availability compared to only a 30% reduction when injected directly into the tumor [120,124].

These contradictions make context based clinical strategies necessary, rather than universally avoiding neutralizing antibodies (NAb). Patient stratification based on integrated NAb features and tumor immune microenvironment is more refined than simple serotype conversion. The future development direction should include: (1) developing quantitative models based on virus characteristics, administration routes, and host immune parameters to predict the impact of NAb; (2) Engineering viruses utilize Fc receptors to target specific parts instead of evading antibody binding; (3) Design a clinical trial on the timing of sequential treatment with checkpoint inhibitors guided by NAb [125].

This evidence synthesis reveals that the relationship between preexisting immunity and oncolytic virus efficacy represents a mechanistic continuum rather than a simple binary effect, with important implications for patient selection and combination strategy design in future clinical protocols (Fig. 2).

#### 4.3.2 Potential Interference of Gut Flora with ICI Response

Gut microbiota profoundly influences clinical responses to ICIs through complex bidirectional metabolic-immune interactions. Clinical observations reveal significant gut flora compositional differences between ICI responders and non-responders; responders typically exhibit higher microbial diversity and enrichment of specific commensal bacteria, including Bifidobacterium species, *Akkermansia muciniphila*, members of the ruminococcaceae family, and *Bacteroides fragilis* [126]. These beneficial microbiota enhance anti-tumor immunity through multiple mechanistic pathways. For example, *Bifidobacterium pseudolongum* secretes inosine to activate T-cell adenosine A2A receptors, promoting Th1 cell differentiation and IFN-γ secretion under interleukin-12 (IL-12) co-stimulation, thereby enhancing anti-PD-1/CTLA-4 efficacy [127]. Specific *Bifidobacterium bifidum* strains facilitate immune microenvironment remodeling by increasing CD8^+^ T-cell infiltration, decreasing regulatory T-cell abundance, and upregulating interleukin-2 (IL-2) and IFN-γ expression [128]. Microbial metabolites function as core regulatory mediators in the host-microbiome interface; short-chain fatty acids (SCFAs) augment CD8^+^ T-cell function through histone deacetylase (HDAC) inhibition, while the tryptophan metabolite indole-3-propionic acid (IPA) sustains T-cell precursor exhaustion by epigenetically modulating histone H3 lysine 27 (H3K27) acetylation in the T-cell factor 7 (Tcf7) gene super-enhancer region, consequently elevating anti-PD-1 response rates in multiple tumor models, including melanoma [126,128,129].

Microbiome modulation presents a double-edged effect: Bacteroidetes phylum enrichment may simultaneously enhance treatment efficacy and increase immune-related enteritis risk, whereas pathogenic bacteria such as *Fusobacterium nucleatum* suppress T-cell activity through recruitment of myeloid-derived suppressor cells (MDSCs) [130]. Antibiotic exposure constitutes a critical confounding factor in clinical outcomes; broad-spectrum antibiotics significantly reduce ICI objective response rates by eliminating beneficial bacteria such as Akkermansia species [131]. Paradoxically, antibiotics may exhibit potentiating effects in specific experimental models through suppression of inflammatory signaling pathways, underscoring the importance of baseline microbiota composition vs. intervention timing [128].

Clinical intervention strategies for microbiome dysbiosis have substantially advanced in recent years, with fecal microbiota transplantation (FMT) transferring microbiome characteristics from responders to non-responders, and engineered bacteria (e.g., SYNB1891 expressing STING agonists) and probiotics (e.g., EDP1503) demonstrating synergistic potential for targeted delivery of immunomodulatory molecules [128]. However, precise regulatory systems and standardized protocols are necessary to optimize the balance between therapeutic efficacy and patient safety in clinical applications.

## 5 Safety Concerns and Management Strategies

### 5.1 Superimposed Toxicity Analysis

#### 5.1.1 Organ-Specific Toxicity of ICI-Associated Immune-Related Adverse Events

The major safety challenge for immune ICI monotherapy arises from organ-specific inflammatory responses. Analyses of clinical trials have shown that the incidence, severity, and chronicity of immune-related adverse events (irAEs) vary significantly across organ systems. The chronic rate of endocrine irAEs is as high as 83%, which usually requires long-term hormone replacement therapy, while the chronic rate of gastrointestinal irAEs is only 16.5% [132].

Key organ-specific manifestations and their distinctive characteristics include:

**Pneumonitis:** Occurs in 2.5%–4% of non-small cell lung cancer patients, with PD-1 inhibitor-associated cases showing higher incidence than PD-L1 inhibitor-induced cases. Severe cases (grade 3–4) manifest as progressive dyspnea, hypoxemia, or respiratory failure, necessitating immediate high-dose glucocorticoid therapy [133]. Approximately 20% of patients require second-line immunosuppressants, with emerging evidence suggesting IL-6 and IL-17 pathway involvement in steroid-resistant cases [132,134].

**Colitis:** Shows significantly higher incidence with CTLA-4 inhibitors compared to PD-1/PD-L1 inhibitors (11.6% vs. 1.3%), clinical features include diarrhea, abdominal pain, and in severe cases, bowel perforation. Endoscopic examination typically reveals diffuse mucosal inflammation, with approximately 35% of cases proving steroid-refractory and requiring anti-TNF-α agents or gut-selective integrin inhibitors [132,135].

**Hepatitis:** Occurs in 2%–6% of patients on PD-1/PD-L1 inhibitor therapy, 4%–9% with CTLA-4 inhibitors, and up to 30% with combination therapy. Histological examination typically reveals periportal CD8^+^ T-cell infiltration and centrilobular necrosis [136–138].

**Endocrine toxicity:** Unlike other irAEs, endocrine toxicities are frequently irreversible, with management focusing on hormone replacement rather than immunosuppression. Early recognition through comprehensive baseline assessment and regular monitoring significantly improves patient outcomes [132,138,139].

#### 5.1.2 Safety Profile of OVs Monotherapy

OVs monotherapy demonstrates a more favorable safety profile, characterized predominantly by low-grade inflammatory responses. Systematic analyses show common OV-related adverse events include fever (56% of studies), chills (40%), injection site pain (15%), flu-like symptoms (27%), and mild liver enzyme elevations (9%), with most classified as grade 1–2 [140].

T-VEC, the first FDA-approved oncolytic viral agent, produced grade 3 fever in only 4% of patients in its phase III trial [141]. Administration route significantly influences the safety profile: intratumoral OVs cause primarily localized reactions, while intravenous OVs may induce transient lymphopenia and systemic inflammatory responses [142].

#### 5.1.3 Potential Safety Interactions in Combination Therapy

The combination of OVs and ICIs may potentiate irAE complexity through multiple mechanisms: First, OVs activate the innate immune system to produce inflammatory factors such as IFN-γ, potentially enhancing PD-L1 expression within the TME and amplifying ICI immunologic effects. Second, the synergistic cytokine effects induced by both therapeutic modalities may elevate the risk of cytokine release syndrome (CRS). Third, OV persistence may sustain immune activation, potentially increasing the risk of irAE chronicity or, in theory, severe events like cytokine release syndrome (CRS). However, available clinical data provides quantification of this risk. In the phase III MASTERKEY-265 trial, grade ≥ 3 TRAEs occurred in 19.1% of patients receiving T-VEC + pembrolizumab vs. 17.7% with pembrolizumab alone, and no grade ≥ 3 CRS events were reported [66]. Similarly, in a phase II trial, grade 3/4 TRAEs were observed in 45% of patients receiving T-VEC + ipilimumab compared to 35% with ipilimumab monotherapy, without specific attribution of severe CRS to the combination [94]. While combination therapy often leads to a higher overall rate of adverse events (frequently additive low-grade events like OV-related fever/chills plus ICI irAEs), current evidence from key trials does not indicate a dramatic increase in new, unexpected grade ≥ 3/4 irAEs or CRS specifically attributable to synergistic hyperactivation compared to ICI monotherapy profiles [140]. Furthermore, CRS remains rarely reported in OV+ICI trials; for instance, no CRS events occurred in a trial of systemic MEDI5395 (NDV) combined with durvalumab [117]. Preclinical models confirm the mechanistic basis, showing OV+ICI can elevate intratumoral cytokines like IFN-γ [71,143], but quantitative data directly comparing severe systemic CRS incidence between monotherapy and combination groups in these models is limited.

As for the treatment restart after the withdrawal of immune checkpoint inhibitor (Irae) in remission, it is suggested to formulate a personalized plan according to the severity of irAEs, recovery status and tumor reaction. In general, recovery treatment can be considered when Irae of grade 1–2 is relieved to ≤grade 1; In the case of grade 3 Irae improved to ≤grade 1, oral vaccine (OV) treatment can be resumed but immune checkpoint inhibitor (ICI) can be permanently stopped; In case of grade 4 or life-threatening irAEs [144,145]. Notably, it is generally recommended to permanently discontinue the two treatments. It is worth noting that since oral vaccines and immune checkpoint inhibitors activate different immune pathways, in theory, sequential administration (e.g., OV followed by ICI), may reduce the risk of superimposed toxicity, but more prospective studies are needed to verify this view.

### 5.2 Safety Optimization Strategies

Optimizing the safety of combination therapy is the key to achieve anti-tumor synergy and reduce adverse reactions. Based on the comprehensive toxicity characteristics analysis and mechanism interaction research, clinical research has established a number of core safety optimization strategies, including the improvement of drug delivery route, virus genetic engineering innovation and treatment scheme optimization. In terms of strategic administration route, intratumoral injection is a precise local administration strategy, which can significantly reduce the distribution of systemic virus and alleviate the intensity of inflammatory reaction. Studies have shown that compared with intravenous administration, intratumoral injection of OV can reduce the incidence of grade 3–4 systemic adverse events by about 35%–62%, while maintaining the effect of local immune activation. For instance, the phase Ib study (NCT02965716) evaluating intratumoral T-VEC combined with pembrolizumab for melanoma treatment reported grade 3 or higher irAEs in only 9.3% of patients, substantially lower than anticipated [146]. In addition, local drug delivery can achieve superior intratumoral virus concentration, which not only enhances direct oncolytic activity but also reduces the exposure of viruses to sensitive organs such as the liver. However, this technique has weak targeting ability for deep or multiple metastatic lesions. Therefore, the academic community is continuously exploring ultrasound/CT-guided injection techniques and nanodetection systems using multi-target molecular strategies [147,148].

Advanced Viral Engineering Strategies: Viral modification represents a rapidly evolving approach to enhance oncolytic virus safety through precise regulation of viral replication and gene expression targeting [16]. MicroRNA regulation technology achieves selective replication by integrating specific targeted microRNA sequences into the viral genome, utilizing the significantly different microRNA expression profiles between normal tissues and malignant tumors. For example, adenoviruses carrying miR-7, miR-122, or miR-148a targeting sequences can effectively inhibit their replication in normal tissues expressing these microRNAs, while still maintaining activity in tumor cells lacking these microRNAs [149,150]. Alternative technical approaches incorporate tissue-specific promoters to control key viral gene expression, as exemplified by prostate cancer-specific adenoviruses controlled by prostate-specific antigen (PSA) promoters that exhibit prostate-selective activity. Safety is further enhanced through deletion of specific viral virulence factors (e.g., HSV-1 γ34.5 gene) or introduction of safety switch genes (e.g., herpes simplex virus thymidine kinase inhibited by acyclovir) [151].

Recent studies have explored the conditional immune-regulatory gene expression system, which enables tumor-specific viruses (OVs) to specifically release immune-regulatory molecules (e.g., IL-12 and GM-CSF) in the tumor-specific microenvironment, thereby promoting local immune activation while reducing the risk of systemic inflammation [152,153]. Treatment scheme optimization: sequential administration has become an effective strategy to alleviate superimposed toxicity in combination therapy.

Traditional synchronous Administration (simultaneous administration of OV and ICI) may cause excessive immune activation, sequential administration (pretreatment of OV before combined treatment) reduces the risk through “progressive immune activation” [65,93]. Experimental evidence demonstrates that OV pretreatment effectively remodels “cold” tumor microenvironments, increases tumor-infiltrating lymphocyte density, and upregulates tumor antigen expression, creating optimal conditions for subsequent ICI therapy. Current research suggests optimal sequential timing involves ICI administration 7–14 days post-viral therapy, when virus-induced innate immune responses have peaked while adaptive immunity remains in early activation phases (NCT03647163) [154]. Additionally, individualized dosing intervals based on dynamic inflammatory marker monitoring (e.g., IL-6, IFN-γ, or peripheral blood CD8^+^/Treg ratios) may optimize safety windows, although studies validating these predictive biomarkers remain ongoing. Dose escalation strategies employing gradual increases from low initial OV doses demonstrate potential for minimizing systemic toxicity, particularly in elderly patients or those with underlying immunodeficiency [153–155]. Risk-Stratified Treatment Protocols: Based on these safety optimization strategies, clinical trials have developed sophisticated risk-stratified treatment algorithms. Low-risk patients (no autoimmune disease history, normal organ function) qualify for standard-dose synchronized or short-interval sequential therapy. Intermediate-risk patients (mild organ insufficiency or well-controlled autoimmune disease) receive delayed ICI therapy following OV pretreatment, preferentially via local administration with potential dose modifications. High-risk patients (multi-system organ insufficiency or active autoimmune disease) may be suitable only for OV monotherapy or ultra-long interval, ultra-low dose exploratory combination regimens [156,157]. Future Research Directions: Emerging research priorities encompass developing individualized dosing regimens based on dynamic viral load monitoring; exploring optimal combination modalities of OVs with various ICIs (anti-CTLA-4 vs. anti-PD-1/PD-L1); investigating safety profiles of triple therapy combinations (e.g., OV + ICI + TLR agonist); and establishing sophisticated early warning systems for irAE detection.

Sequential administration mitigates additive toxicity while maximizing immune synergy. Pharmacokinetic studies reveal that intratumoral OVs achieve peak titers (10^9^–10¹¹ PFU/g) within 24–48 h, inducing innate immune activation (IFN-γ↑, DC maturation) by 72 h, whereas intravenous OVs show delayed tumor accumulation (<10^7^ PFU/g) and attenuated cytokine responses [158]. Consequently, ICI administration should align with the peak of OV-primed T-cell exhaustion: for IT delivery, optimal ICI timing is Day 7–14 post-OV, coinciding with PD-1 upregulation on tumor-infiltrating CD8^+^ T cells; for IV delivery, delayed ICI initiation (Day 10–21) compensates for slower immune priming [105,154]. Clinical data from NCT02263508 validate this approach, demonstrating significantly higher ORR with Day 7 pembrolizumab after IT T-VEC vs. concurrent dosing (48.6% vs. 41.3%) [154]. Future protocols should integrate dynamic biomarkers (e.g., serum IFN-γ, CD8^+^/Treg ratios) to personalize this window (Table 3).

**Table 3:** Pharmacokinetics (PK): route-dependent biodistribution

Parameter	Intratumoral (IT)	Intravenous (IV)	References
Peak viral titer	High (10^9^–10^11^ PFU/g tumor)	Low (10^6^–10^7^PFU/g tumor)	[105,158]
Systemic exposure	Minimal (<1% blood circulation)	High (rapid liver/spleen sequestration)	[103,106]
Tissue penetration	Limited to injected lesion	Potential for metastatic sites	[103,105]
Neutralization	Low (bypasses serum antibodies)	High (neutralizing antibody clearance)	[118,124]
Half-life (t/2)	Days (sustained replication)	Hours (rapid clearance)	[143,158]

In conclusion, the toxicity management related to combination therapy is changing from passive intervention to active prevention strategy, through the integration of local drug delivery methods, engineering virus design innovation and optimization of drug delivery scheme. This comprehensive framework provides a solid foundation for improving the safety of clinical practice, while maximizing the therapeutic effect.

## 6 Future Directions and Challenges

### 6.1 Novel OVs Design

Genetic engineering of OVs to deliver immunomodulatory molecules represents a paradigmatic advancement in optimizing tumor immunotherapy, particularly when synergistically integrated with ICIs. Preclinical investigations demonstrate that this strategic approach augments direct oncolytic activity while achieving sustained therapeutic efficacy through comprehensive remodeling of the TME and activation of T cell-mediated anti-tumor immunity [159]. Notably, cytokine-armed OVs exhibit superior immunomodulatory potential. For instance, the measles virus variant MeVac FmIL-12, engineered to encode an IL-12 fusion protein, induces complete tumor regression in 90% of immunocompetent MC38cea mouse models through orchestrated Th1-directed immune responses, upregulation of IFN-γ and TNF-α, and coordinated activation of NK and CD8^+^ T cell populations [160]. Similarly, T-VEC, an FDA-approved oncolytic herpesvirus expressing GM-CSF, potentiates antigen-presenting cell maturation and subsequently activates tumor-specific T-cell responses [161].

Despite their promise, engineered OVs face significant challenges, including stringent viral vector capacity limits. HSV-1 vectors typically tolerate <8 KB of exogenous DNA without compromising replication efficiency, while adenoviruses are restricted to ≈8 KB inserts and vaccinia viruses exhibit reduced replication kinetics with inserts >15 KB. This constrains the co-expression of large immunomodulators (e.g., full-length antibodies ≈ 4–5 KB). To address these limitations, innovative strategies have emerged:

Compact transgene designs (e.g., single-chain antibodies/scFv ≈ 0.8–1 KB replacing full-length mAbs), exemplified by ONCR-177 (HSV-1) expressing anti-PD-1/CTLA-4 scFvs + TNFα within a single genome [70]. Self-cleaving peptide systems (e.g., P2A/T2A) enabling multicistronic expression from a single promoter, as utilized in VG161 (HSV-1) for simultaneous delivery of IL-12, IL-15, and PD-L1 blocker [73]. Tumor-specific promoters (TSPs) and logic-gated circuits (e.g., Lokon Pharma Adenovirus 703 (LOAd703) adenovirus driving IL-2 and 4-1BB Ligand (4-1BBL) via Δ24 E1A-TSP) ensuring spatially restricted transgene activation [92].

These approaches collectively enhance therapeutic efficacy by enabling synergistic payload combinations (e.g., cytokines + checkpoint inhibitors) while maintaining safety through tumor-targeted expression. For multi-gene regimens exceeding single-vector capacity, dual-vector systems or heterologous prime-boost strategies (e.g., OV-cytokine followed by OV-scFv) show preclinical success in overcoming immunosuppressive TMEs, though they require optimization of delivery kinetics to mitigate immune clearance risks [159].

Combination therapeutic regimens substantively amplify treatment efficacy. The integration of MEK inhibitors with OVs, exemplified by trametinib in conjunction with T-VEC, demonstrates synergistic enhancement of melanoma cell cytotoxicity *in vitro*, attenuated tumor progression *in vivo*, and prolonged survival outcomes in murine models. This therapeutic approach increases CD8^+^ T cell infiltration, promotes antigenic epitope spreading, and upregulates pro-inflammatory gene expression profiles. Notably, triple therapy combining T-VEC, trametinib, and anti-PD-1 agents achieves near-complete tumor eradication, illustrating the transformative potential of multi-target strategies in overcoming monotherapy limitations [143].

OVs encoding ICIs also demonstrate therapeutic promise by neutralizing immunosuppressive TME signals, inducing tumor regression, and establishing long-term immune memory. However, challenges persist: (1) viral vector capacity constraints necessitate optimization of immunomodulatory gene size and number [162]; (2) spatiotemporal control of immunomodulatory factor expression must align with viral replication kinetics (e.g., differing replication rates between MeVac FmIL-12 and MeVac GM-CSF) [160]; (3) differences in immune systems and TME heterogeneity limit preclinical translatability.

Future directions include:

① Conditional expression systems (e.g., tumor-specific promoters) for precise spatiotemporal control of immunomodulators.

② Multifunctional OVs co-expressing complementary factors to synergize immune pathways.

③ TME-restricted activity via tumor-specific protease cleavage sites to minimize systemic toxicity.

④ Artificial Intelligence (AI)-driven predictive models to guide personalized OV design and dosing based on tumor immune profiles.

Next-generation OVs must prioritize tumor-specific immune activation. For example, logic-gated designs (e.g., protease-responsive IL-15 in LH05) or dual-gene strategies (e.g., CD40L + 4-1BBL) can amplify Antigen-Presenting Cell (APC)—T cell crosstalk while reducing off-target effects [163]. Despite challenges—viral genome capacity, tumor heterogeneity, immunosuppressive TME components, and clinical translation hurdles (delivery efficiency, manufacturing standardization)—integrating CRISPR/Cas9, AI, and humanized models may enable “smart” OVs platforms [164–166]. Combining OVs with ICIs, co-stimulatory molecules, and small-molecule inhibitors could transform immunologically “cold” tumors into “hot” ones, revolutionizing solid tumor treatment [38].

### 6.2 Triple Combination Strategies: OVs, ICIs, and Complementary Therapies

Although the combination of OVs and ICIs has made significant progress, the heterogeneity of TME and acquired drug resistance mechanism still restrict the clinical efficacy. Contemporary research work focuses on the development of triple therapy and the integration of complementary therapies to overcome the existing limitations and establish a lasting anti-tumor immune response.

Radiotherapy-inclusive combinations demonstrate remarkable synergy by inducing immunogenic cell death while simultaneously modifying the immunosuppressive TME. Liu and colleagues demonstrated that Preclinical studies suggest that combining radiotherapy with HSV-1 and anti-PD-1 antibodies may enhance antitumor immunity through IL-1α-dependent CD8^+^ T cell activation. In murine models, this triple regimen significantly reduced local recurrence risk (HR = 0.32, *p* < 0.01) and suppressed distant metastasis [167,168]. Early clinical observations suggest translational potential: In a case report, progression free survival of 44 months was recorded in PD-1 resistant squamous cell carcinoma patients receiving this combination therapy [94]. However, robust validation through larger Phase II/III trials is imperative to confirm efficacy and safety in heterogeneous human populations. Notably, interspecies differences in TME dynamics (e.g., immune cell infiltration patterns, viral replication efficiency) necessitate cautious extrapolation of preclinical findings to clinical practice.

Epigenetic regulators (HDAC/DNMT inhibitors) enhance therapeutic effects through multiple mechanisms, including upregulation of MHC and optimization of antigen presentation [169]. The ability of these agents to increase tumor-infiltrating T cell populations by 65% while reducing tumor volume by 82% highlights their potential in creating a more responsive TME [170–172].

Targeted therapy shows unique promise in mutation-driven cancers: when BRAF inhibitors are combined with oncolytic herpes simplex virus (HSV) and checkpoint inhibitors, the complete response rate in BRAF-mutated thyroid cancer reaches 93%, accompanied by significant changes in immune cell activation markers and interferon signaling pathways [132,173].

These therapies aim at different levels of tumor immune relationship, and jointly build a complex treatment network by generating synergistic effects beyond a single treatment scheme. However, the current research has methodological limitations, including differences in clinical trial design, insufficient sample size and insufficient standardization of biomarkers, which have brought major challenges to the comprehensive understanding and optimization of this combination therapy. The development trend in this field is increasingly towards advanced synthetic biological methods, which can design intelligent virus vectors to recognize specific TME and activate the corresponding signal pathways.

These adaptive strategies are particularly promising in metastatic diseases, because different metastatic sites show different characteristics and require customized treatment interventions.

### 6.3 Precision Treatment Strategies

The molecular characteristics and temporal dynamics of the immune microenvironment exert a decisive influence on OV-ICI therapeutic efficacy. For example, isocitrate dehydrogenase 1 (IDH1) mutations in gliomas inhibit interferon regulatory factor 3/7 (IRF3/7) expression, thereby attenuating type I interferon responses [174,175]. Comprehensive multi-omics analytical approaches (integrating genomics, immunogenomics, and transcriptomics) contribute to the development of precisely tailored oncolytic viral vectors [176]. Future directions for OV-ICI combination precision oncology include advanced synthetic biology approaches predicated on systems biology principles. These strategies aim to engineer intelligent OVs capable of recognizing specific tumor microenvironmental conditions, activating context-dependent signalling pathways, and modulating gene expression patterns to optimize tumor eradication. These adaptive approaches hold particular promise for metastatic diseases, wherein distinct metastatic sites frequently exhibit divergent phenotypic characteristics and consequently require individualized therapeutic interventions.

### 6.4 Methodological Limitations in Existing Literature

Although OV-ICI combination therapies show great promise in the existing literature, their clinical translation is still constrained by multiple limitations. Heterogeneity in clinical trial design poses the primary obstacle, with inconsistencies in patient stratification criteria, dosing regimens, and endpoint definitions significantly hampering inter-trial comparability. The diversity of TMB thresholds defining “cold” or “hot” tumors, and the insufficient sample size of early-stage studies have combined to limit the quantitative resolution of therapeutic synergies and the establishment of reliable predictive biomarkers [177,178]. Deficiencies in preclinical models further hinder the translational process. Animal models struggle to mimic the complexity of the human TME, such as immune cell diversity and virus-host co-evolutionary dynamics, while immunodeficiency models overestimate the efficacy of OVs due to interspecies differences in intrinsic immune signaling, and an over-focus on *in situ* tumors ignores the unique immunosuppressive properties of the metastatic niches [179–181].

Despite the identification of potential predictive markers such as PD-L1 expression levels and CD8^+^ T-cell infiltration density, their clinical application is still limited by the lack of biomarker standardisation. Heterogeneity in quantification methods (e.g., differences in assay platforms, varying interpretation criteria) and dynamic fluctuations in the treatment timeline combine to weaken the reliability of markers; and the lack of validated composite biomarkers integrating multidimensional TME features further hampers the development of precise individualised treatment strategies [182–185].

Future research is urgently needed to break through the following key bottlenecks:

1. Establish a unified clinical trial design specification and adopt a standardised biomarker definition and stratification method; 2. Develop a humanised advanced model of the immune system to accurately mimic human tumor-immune interactions; 3. Implement a dynamic biomarker monitoring technology to capture the temporal evolution of treatment response; 4. Build a multi-omics integrated analysis platform to systematically analyse the construct an integrated multi-omics analysis platform to systematically analyse the tumor-immunity dynamic signature profile.

Despite these challenges, the remarkable convergence of mechanistic understanding, accumulating clinical evidence, and technological innovation positions OV-ICI combination therapy as a transformative paradigm in cancer immunotherapy. The ability to convert immunologically “cold” tumors into “hot” ones through virally induced inflammation, coupled with checkpoint inhibition to sustain T cell responses, represents a fundamental shift in approaching treatment resistance.

With the development of personalized immunotherapy under the guidance of virus engineering platforms, the success of this combination method will depend on strict biomarker validation, optimization of treatment plans, and continuous innovation in virus vector design.

With these advances, the OV-ICI combination is expected to redefine the treatment criteria for multiple cancer indications, ultimately improving the prognosis of patients with previously limited treatment options. Despite methodological limitations, OV-ICI combination therapy represents a transformative approach to immunotherapy. It has the ability to transform immune “cold” tumors into “hot” tumors through viral inflammation, coupled with checkpoint inhibition that maintains T cell responses, providing a new approach for treating drug resistance. With the maturity of precision medicine and virus engineering platforms, the field is moving towards personalized protocols. Success depends on biomarker validation, treatment sequence optimization, and viral vector innovation. Through standardized clinical trial protocols and enhanced translational preclinical models, the OV-ICI combination approach has significant potential to redefine treatment standards for multiple cancer indications, ultimately improving patient outcomes with limited efficacy of traditional immunotherapy methods. Despite methodological limitations, OV-ICI combination therapy represents a transformative immunotherapeutic paradigm. Its capacity to convert immunologically “cold” tumors to “hot” through viral inflammation, coupled with checkpoint inhibition sustaining T cell responses, provides a novel approach to treatment resistance. The field advances toward personalized protocols as precision medicine and viral engineering platforms mature. Success depends on biomarker validation, treatment sequence optimization, and viral vector innovation. With standardized clinical trial protocols and enhanced translational preclinical models, OV-ICI combination approaches possess significant potential to redefine therapeutic standards across multiple cancer indications, ultimately improving patient outcomes in contexts where conventional immunotherapeutic approaches have demonstrated limited efficacy.

## Data Availability

Not applicable.

## Abbreviations

ADCC: Antibody-Dependent Cellular Cytotoxicity
Ad5: Adenovirus Serotype 5
AI: Artificial Intelligence
APC: Antigen-Presenting Cell
ATM: Ataxia Telangiectasia Mutated
ATR: ATM and Rad3-related
ATP: Adenosine Triphosphate
B2M: β2-Microglobulin
BRAF: B-Raf Proto-Oncogene
C3b: Complement Component 3b
C5b: Complement Component 5b
CAR: Coxsackie-Adenovirus Receptor
CAR-T: Chimeric Antigen Receptor T Cell
CCL5: C-C Motif Chemokine Ligand 5
CD40L: CD40 Ligand
cGAS: Cyclic GMP-AMP Synthase
CR: Complete Response
CRISPR: Clustered Regularly Interspaced Short Palindromic Repeats
CRS: Cytokine Release Syndrome
CTLA-4: Cytotoxic T-Lymphocyte-Associated Protein 4
CTLs: Cytotoxic T Lymphocytes
CXCL1: C-X-C Motif Chemokine Ligand 1
CXCL2: C-X-C Motif Chemokine Ligand 2
CXCL10: C-X-C Motif Chemokine Ligand 10
CXCL11: C-X-C Motif Chemokine Ligand 11
DAMPs: Damage-Associated Molecular Patterns
DCs: Dendritic Cells
DNMT: DNA Methyltransferase
DNMT3B: DNA Methyltransferase 3 Beta
ECM: Extracellular Matrix
EGFR: Epidermal Growth Factor Receptor
eIF2α: Eukaryotic Initiation Factor 2α
EV-A71: Enterovirus A71
FMT: Fecal Microbiota Transplantation
GM-CSF: Granulocyte-Macrophage Colony-Stimulating Factor
H3K4me3: Histone H3 lysine 4 trimethylation
H3K27: Histone H3 lysine 27
HCC: Hepatocellular Carcinoma
HDAC: Histone Deacetylase
HER2: Human Epidermal Growth Factor Receptor 2
HMGB1: High Mobility Group Box 1
HNSCC: Head and Neck Squamous Cell Carcinoma
HPV: Human Papillomavirus
HSPG: Heparan Sulfate Proteoglycan
HSV: Herpes Simplex Viruses
HSV-1: Herpes Simplex Virus Type 1
HVEM: Herpesvirus Entry Mediator
HVR: Hypervariable Region
ICIs: Immune Checkpoint Inhibitors
ICD: Immunogenic Cell Death
IDH1: Isocitrate Dehydrogenase 1
IFN-γ: Interferon Gamma
IFN-I: Type I Interferon
IgG1: Immunoglobulin G1
IgG3: Immunoglobulin G3
IgG4: Immunoglobulin G4
IL-2: Interleukin-2
IL-12: Interleukin-12
IL-15: Interleukin-15
IL-23: Interleukin-23
IPA: Indole-3-Propionic Acid
irAEs: Immune-Related Adverse Events
IRF3: Interferon Regulatory Factor 3
IRF7: Interferon Regulatory Factor 7
LAG-3: Lymphocyte-Activation Gene 3
LOAd703: Lokon Pharma Adenovirus 703
MDSCs: Myeloid-Derived Suppressor Cells
MEK: Mitogen-Activated Protein Kinase Kinase
MHC-I: Major Histocompatibility Complex Class I
M-MDSC: Monocytic Myeloid-Derived Suppressor Cells
NAbs: Neutralizing Antibodies
NECTIN-1: Nectin Cell Adhesion Molecule 1
NK cells: Natural Killer Cells
NR4A: Nuclear Receptor subfamily 4 group A
NSCLC: Non-Small Cell Lung Cancer
ORR: Objective Response Rate
OS: Overall Survival
OVs: Oncolytic Viruses
P2A: Porcine teschovirus-1 2A
PD-1: Programmed Death-1
PD-L1: Programmed Death-Ligand 1
PFS: Progression-Free Survival
PFU: Plaque-Forming Units
PKR: Protein Kinase R
PMN-MDSC: Polymorphonuclear Myeloid-Derived Suppressor Cells
PSA: Prostate-Specific Antigen
SCARB2: Scavenger Receptor Class B Member 2
SCFAs: Short-Chain Fatty Acids
scFv: Single-Chain Variable Fragment
STAT5: Signal Transducer and Activator of Transcription 5
STING: Stimulator of Interferon Genes
T2A: *Thosea asigna* virus 2A
TAAs: Tumor-Associated Antigens
TAP: Transporter Associated with Antigen Processing
TCR: T-Cell Receptor
Tcf7: T-cell factor 7
Teff: Effector T Cells
TGF-β: Transforming Growth Factor Beta
Th1: T Helper 1 Cells
TILs: Tumor-Infiltrating Lymphocytes
TIM-3: T-cell Immunoglobulin and Mucin-domain containing-3
TLR4: Toll-Like Receptor 4
TLR9: Toll-Like Receptor 9
TMB: Tumor Mutational Burden
TME: Tumor Microenvironment
TNBC: Triple-Negative Breast Cancer
TNF-α: Tumor Necrosis Factor Alpha
TOX: Thymocyte selection-associated high mobility group box
TRAEs: Treatment-Related Adverse Events
Tregs: Regulatory T Cells
TSPs: Tumor-Specific Promoters
T-VEC: Talimogene Laherparepvec (Oncolytic Herpes Simplex Virus-1 expressing GM-CSF)
VVs: Vaccinia Viruses
4-1BBL: 4-1BB Ligand
